# The impact of metallothionein-II on microglial response to tumor necrosis factor-alpha (TNFα) and downstream effects on neuronal regeneration

**DOI:** 10.1186/s12974-018-1070-3

**Published:** 2018-02-22

**Authors:** Jacqueline Y. K. Leung, William R. Bennett, Anna E. King, Roger S. Chung

**Affiliations:** 1 Wicking Dementia Research and Education Centre, College of Health and Medicine, University of Tasmania, Medical Science Precinct 1, 17 Liverpool Street, Hobart, TAS 7000 Australia; 2 Department of Biomedical Sciences, Faculty of Medicine & Health Sciences, Macquarie University, 2 Technology Place, Sydney, NSW 2109 Australia

**Keywords:** Metallothionein, Microglia, Low-density lipoprotein receptor-related protein-1, Low-density lipoprotein receptor-related protein-2, Axon regeneration, Traumatic brain injury

## Abstract

**Background:**

The extracellular environment plays an important role in supporting the regeneration of axons after injury. Metallothionein-II (MTII) is a metal-binding protein known for its neuroprotective effect by directly stimulating the growth of axons after injury. Previous studies have shown that MTII also modulates the response of astrocytes and microglia after injury. However, a detailed analysis describing how MTII modulates the interaction between microglia and neurons is lacking.

**Methods:**

We introduced fluorescently labelled MTII into the cortex at the time of needlestick injury to investigate the cellular uptake of MTII using immunohistochemistry with antibodies against cell-type-specific markers. The role of MTII in modulating the effect of microglia on axon outgrowth following an inflammatory response is further investigated using a co-culture model involving primary rodent microglia pre-treated with TNFα and primary rodent cortical neurons. The axon lengths were assessed 24 h after the plating of the neurons onto treated microglia. We also utilised siRNA to knockdown the expression of LRP1, which allows us to investigate the role of LRP1 receptors in the MTII-mediated effect of microglia on axon outgrowth.

**Results:**

Fluorescently labelled MTII was found to be associated with neurons, astrocytes and microglia following injury in vivo. Microglia-neuron co-culture experiments demonstrated that exogenous MTII altered the response of microglia to TNFα. The neurons plated onto the TNFα-stimulated microglia pre-treated with MTII have shown a significantly longer axonal length compare to the TNFα-stimulated microglia without the MTII treatment. This suggested that MTII reduce cytokine-stimulated activation of microglia, which would ordinarily impair neurite outgrowth. This inhibitory effect of MTII on activated microglia was blocked by siRNA-mediated downregulation of LRP1 receptor expression in microglia, suggesting that MTII acts via the LRP1 receptor on microglia.

**Conclusions:**

This study demonstrates that exogenous MTII acts via the LRP1 receptor to alter the inflammatory response of microglia following TNFα stimulation, providing a more supportive environment for axon growth.

## Background

Glial cells play an important role in maintaining the health of neurons and particularly following injury to the central nervous system (CNS). In this regard, astrocytes and microglia contribute actively to the formation of the glial scar, which is strongly inhibitory to axon regeneration [1, 2]. Chondroitin sulphate proteoglycans (CSPGs) are one of the major molecules expressed by glia that contribute to the inhibitory environment formed by the glial scar. Modulating this inhibitory environment using agents such as chondroitinase ABC has been shown to improve the regeneration outcome after injury [3]. Chondroitinase ABC enzymatically digests CSPG, which improves neuron survival, axonal growth as well as synaptic plasticity following middle cerebral artery occlusion [4]. These, and many other studies [5–7], demonstrate that improving the extracellular environment plays a vital role in improving axon regeneration after injury. Apart from CSPG, myelin-associated inhibitors such as myelin-associated glycoprotein (MAG), Nogo and Oligodendrocyte myelin glycoprotein (OMgp) are also present in the glial environment and have a significant role in inhibiting axon outgrowth as well as plasticity [6, 8, 9, 10]. Soluble inhibitory cues such as semaphorins are also recognised as having an important contribution to the inhibitory environment after CNS injury [9, 10]. In contrast to factors that inhibit neurite outgrowth, other factors have been identified to promote neurite outgrowth. These include neurotrophic factors (such as nerve growth factors, brain-derived neurotrophic factor) as well as several extracellular matrix attractive guidance molecules that are present at the injury site [6, 9, 10]. The balance between the inhibitory and promoting factors in the injury site are important in determining the outcome of axon regeneration after injury.

Metallothionein I/II represents an unusual family of metal-binding proteins; both MTI and MTII isoform share a very similar structure as well as have a range of neurotrophic properties. Our group has previously demonstrated that extracellular MTII directly interacts with axons and improves axon outgrowth via the low-density lipoprotein receptor 2 (LRP2, also called megalin) [9, 10]. Apart from its direct actions on neurons, we have also identified a potential role of MTI/II in modulating the trophic environment provided by the glial response to promote neuron regeneration [11, 12]. The use of transgenic animals (both MTI/II knockout and MTI overexpression mice) has further demonstrated that MTI/II has an active role in modulating the microglial response after injury. When a freeze-lesion cortical injury (a localised cortical injury caused by applying dry ice directly on the skull) was performed on the mice with MTI overexpression, this results in a significant decrease in the number of activated microglia near the freeze lesion site in comparison to the wild-type control mice [13]. In contrast, the freeze-lesion cortical injury in MTI/II-knockout mice results in an increase in microglia/macrophage infiltration and activation as well as a delayed in wound repair process when compared to the wild-type control mice [14]. Similar results have also been reported in rodent needlestick injury model, where the administration of extracellular MTII into the injury site improved regenerative sprouting of axons [15].

Together, these studies suggested that both MTI and MTII have an active role in modulating the inflammatory response as well as wound healing after injury. Mechanistic studies performed in our laboratory have now begun to reveal how MTII modulates the neuroinflammatory response of microglia following traumatic brain injury. We have previously shown that MTII has a direct effect in decreasing the expression of quinolinic acid (QUIN) in microglia along the injury tract in the rat cortical needlestick injury model [12]. QUIN is one of the neurotoxic intermediates of the kynurenine pathway that is significantly upregulated in inflammatory microglia and macrophages [16]. In cultured microglia, MTII treatment led to a decrease in QUIN expression upon stimulation with the pro-inflammatory interferon-γ (IFN-γ) [12]. In the current study, we have explored the mechanism of MTII signalling in microglia and the subsequent impact upon neuronal regeneration. We show that MT modulates the inflammatory response of tumor necrosis factor (TNF)-α-treated microglia via the LRP1 receptor and that this action of MTII upon microglia causes them to become a more permissive substrate to support axonal outgrowth in vitro.

## Methods

### MetallothioneinII protein labelling

The MTII protein used in these studies was native rabbit Zn_7_-MT-IIA (the major brain-expressed mammalian MTII isoform) purified by high-performance liquid chromatography (Bestenbalt LLC, Estonia). The MTII was N-terminally tagged using the AlexaFluor® 488 Protein Labelling Kit (Invitrogen) in accordance with the accompanying protocols. During the column fractionation of labelled protein, all of the eluted fractions were collected including the unincorporated dye (the second eluted fluorescent peak). The fractions collected from the first eluted fluorescent peak contained the MTII conjugated with the AlexaFluor 488 tag and is hereafter referred to as MT_488_, while the unincorporated dye peak was used as the vehicle treatment and is referred to as A_488_.

### Focal cortical needlestick injury in adult rats

Focal needlestick injuries to the Par1 somatosensory region of the cortex were performed as described previously [15]. Briefly, a 1 μL 25-gauge Hamilton syringe was used to stereotactically deliver the different treatments: MT_488_ (2 mg/mL) and A_488_ into the cortex (*n* = 4 per treatment). The volume of injection for all treatments was 1 μL. The injury was induced by inserting the Hamilton syringe to a depth of 1.5 mm into the cortex. After leaving the syringe in place for 5 min, 0.5 μL of the treatment was injected into the injury site. After a further 5 min, the needle was first raised by 0.5 mm and the remaining 0.5 μL of the treatment was then injected into the cortex. Absorbent Gelfoam (soaked with 5 μL of the treatment solution, either MT_488_ or A_488_) was placed on the surface of the cortex, and the site of injury was sutured closed.

### Perfusion and processing of the brain injury site

The animals were sacrificed 4 days after injury by an intraperitoneal injection of pentobarbitone (0.4 mg/g of animal weight) to induce a deep anaesthesia followed by cardiac perfusion with 4% paraformaldehyde. The brain was then excised and post-fixed in 4% paraformaldehyde + 4% sucrose solution in phosphate buffer saline (PBS) for 24 h. Brains were cryoprotected in 10 and 30% sucrose solutions (24 h incubation at 4 °C for each solution) prior to cryosectioning. The brain samples were cryosectioned at 16 μm in a horizontally sectioned plane cut parallel to the superior surface of the brain using the Leica CM1850 Cryostat, and serial sections were then mounted on APTS-treated microscopic slides. For a consistent comparison between treatments, the sections were obtained at similar depth across all treatments. Sections that were collected and processed for immunohistochemistry labelling were located from approximately 60 to 540 μm from the top of the cortex, corresponding to layer II and III of the supragranular layer of the somatosensory cortex.

### Immunohistochemistry on tissue sections

Brain sections were incubated with the following primary antibodies: Mouse (ms)-SMI312 (1:1000, Covance), ms-MT (1:1000, Dako), Rabbit (rb)-Iba-1 (1:500, Wako), Rb- Glial fibrillary acidic protein (GFAP, 1:1000, Dako). All primary antibodies used were diluted in 0.03% Triton-X/PBS and incubated at 4 °C overnight. Following the incubation, the primary antibodies were washed three times with PBS. The sections were then incubated with the respective secondary antibodies: AlexaFluor-594 or -488 anti-mouse (1:1000, Invitrogen), AlexaFluor-594 or -488 anti-rabbit (1:1000, Invitrogen). The secondary antibodies were diluted in PBS and applied to sections for 1 h at room temperature prior to incubation with Nuclear Yellow (1 μg/mL, Molecular Probes) for 5 min. The sections were mounted using fluorescent mounting agent (Dako).

### Image acquisition

Images were captured using either an epifluorescence microscope (Olympus BX50) with mounted camera (CoolSNAP HQ^2^ camera) or confocal microscope (Zeiss LSM 510). In some cases, a confocal *Z*-axis series of digital images was captured through the injury site (minimum 0.5 μm *Z*-axis spacing).

### Purified rodent microglia cultures

Purified rodent microglia cultures were prepared as described previously [12]. Briefly, mixed glial cultures were prepared from the cortex of Sprague Dawley postnatal day 2 rat pups in 75 cm^2^ tissue culture flasks, and maintained at 37 °C with 5% CO_2_. The culture medium (Dulbecco’s modified eagle medium, DMEM, from Sigma + 10% Fetal calf serum from Gibco + 1% penicillin-streptomycin-amphotericin B solution from Gibco) was replaced on the following day with fresh medium, and culture was maintained to confluence (around 2 weeks after initial plating). Upon confluence, the culture medium was changed to serum-free medium (DMEM + 1% penicillin-streptomycin-amphotericin B solution), and flasks were horizontally shaken at 250 rpm for 30 min, which was sufficient to dislodge the microglia in these mixed glial cultures. The culture medium containing the microglia was collected, and microglia were pelleted by centrifugation at 500×*g* for 10 min. Microglia were re-suspended and then plated at the required number into 24-well plates (15,000 cells per well containing coverslips pre-coated with 0.025% poly-L-lysine). Microglia were maintained in serum-free media until confluence.

### Neuron and microglia co-culture

Microglia were collected and plated onto glass coverslips in serum-free media as described previously. Two days after the plating of microglia, the cultures were incubated with the respective treatment accordingly for 24 h in serum-free media. These treatments were 10 ng/mL TNFα (Abcam) with or without MTII (1 μg/mL) and saline (vehicle treatment). Cortical neuron cultures were prepared as reported previously from embryonic day 17 Sprague-Dawley rats [15]. In order to prevent the effect of the extracellular MTII or TNFα on the growing neurons, the media of the microglia culture was changed to neuron media (Neurobasal™ medium supplement with B-27 supplement, 0.1 mM L-glutamine and 10% fetal bovine serum from Gibco) prior to the addition of cortical neurons on top of the microglia at cell density of 2 × 10^4^ cells per well. Co-cultures were incubated for 24 h followed by fixation with 4% paraformaldehyde for 15 min at room temperature.

### siRNA knockdown of LRP1 receptors

We then employed pre-designed siRNA (ThermoFisher Scientific) to selectively knockdown the LRP1 receptors in microglia cultures as described previously [17]. The knockdown of LRP1 receptors expression in microglia was also confirmed via Western blotting as described previously [17]. The microglia were first collected (as above) and resuspended in 1 mL of serum-free media containing 100 nM of siRNA against LRP1 (from ThermoFisher Scientific). The microglia were plated into 24-well plates (procedure stated as above) and incubated for 1 h at 37 °C 5% CO_2._ The concentration of the siRNA was diluted down to 10 nM in the well by adding in the additional culture media; the cultures were then returned back to the incubator.

### Immunocytochemistry on culture

Fixed cultures were incubated with primary antibodies (ms-SMI312 used at 1:1000, Covance; rb-LRP1 used at 1:1000, Sigma-Aldrich; rb-megalin used at 1:1000, Santa Cruz Biotechnology) diluted in 0.03% Triton-X/PBS for 24 h at 4 °C. The primary antibodies were then washed three times in PBS (5 min per washes on shaker at room temperature). The coverslips were incubated with secondary antibodies (AlexaFluor-488 anti-mouse used at 1:1000, Invitrogen) in PBS for 1 h at room temperature on a shaker. All cultures were labelled with Nuclear Yellow (1 μg/mL, 5 min) followed by two times washes with PBS. Cultures were mounted on glass slides using fluorescent mounting agent (Dako).

### Imaging and statistical analysis

Digital images (five images per coverslips) of SMI312 immunolabelled axons were acquired on a fluorescence microscope (Leica DM-LB2) with an Olympus Magnifier cooled-CCD camera. Lengths of axons were measured using ImageJ (National Institute of Health) software. The data were analysed using the Student *t* test, with *p* < 0.05 considered as significant. For MTII and TNFα experiment, *n* = 4 experimental cultures were prepared and one coverslip per culture per treatment group was used. For LRP1 siRNA experiment, *n* = 4 experimental cultures were prepared and two coverslips per culture per treatment group were used.

## Results

### Exogenous metallothionein-II is associated with neuron, astrocytes and microglia after needlestick injury

Our previous studies have shown that MTII can promote neurite outgrowth of cultured cortical neurons [15], through a mechanism involving LRP receptors and downstream MAPK signalling [9, 10]. We have also shown in cell culture that MTII can modulate the astrocytic response to neurotrauma, leading to a more permissive environment to allow the extension of axons [11]. Similarly, MTII can act upon cultured microglia to suppress their inflammatory activation following cytokine stimulation. When administered by direct injection into the site of a cortical needlestick injury, MTII modulates glial reactivity and promotes regenerative axonal sprouting [15]. What remains unclear is which cell type/s are directly modulated by MTII treatment in vivo and the sequence of cellular events elicited by MTII that ultimately contributes to improved neural regeneration. To investigate this, we utilised fluorescently labelled MTII (MT_488_), which was introduced into the cortex via the needlestick injury. Tissue was harvested at 4 days post-injury prior to immunohistochemistry.

We first confirmed that the administration of MT_488_ produces the same neuroprotective effect that has been reported previously. Accordingly, it was found that MT_488_ reduced the severity of the experimental injury, clearly observable by immunohistochemistry of axonal structures (using antibody against SMI312) (results not shown). This demonstrates that the MT_488_ displays similar neurotrophic activity to unlabelled MTII [15].

**Fig. 1:**
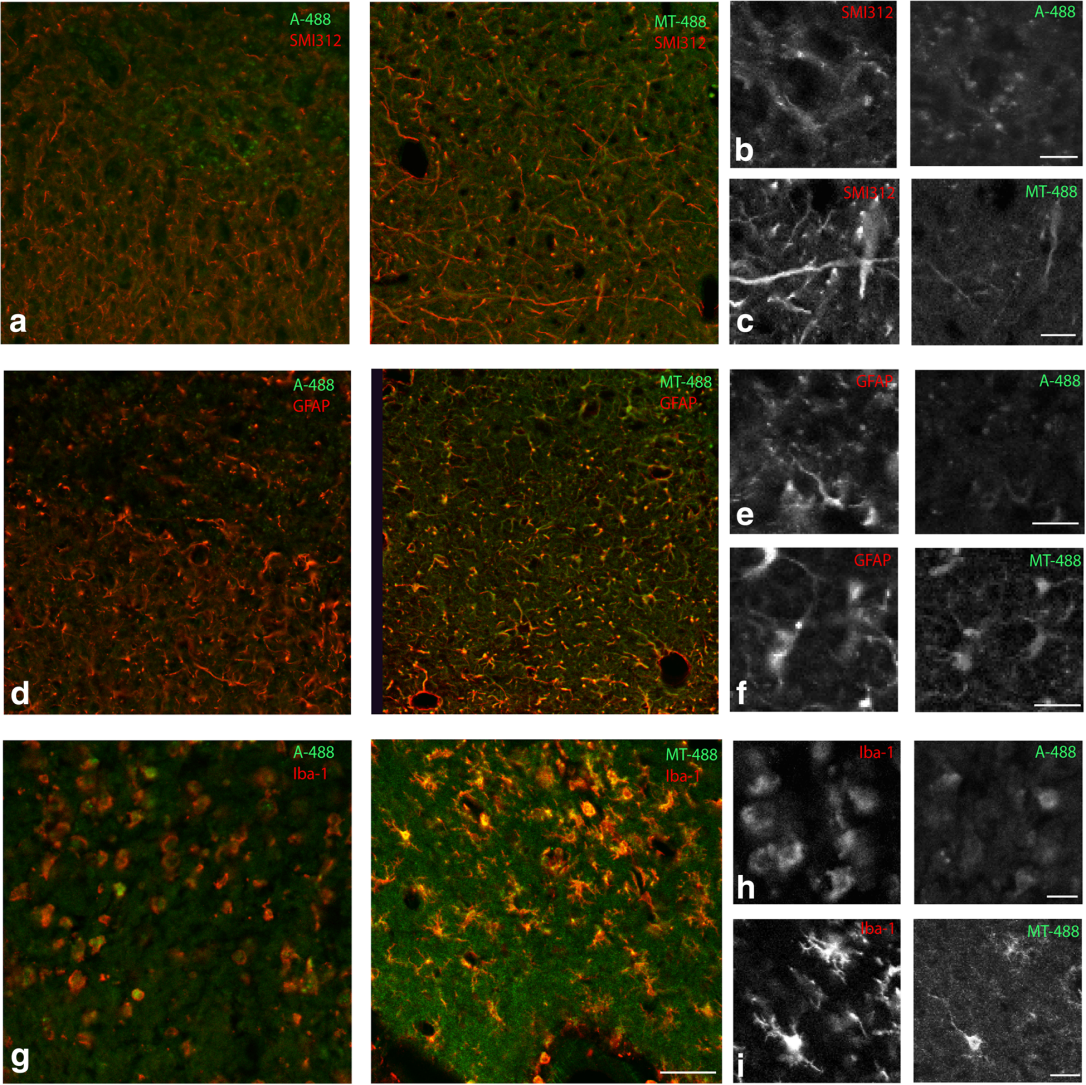
Fluorescently labelled Metallothionein I/II (MT_488_) is taken up by neurons, astrocytes and microglia after needlestick injury. Immunohistochemistry using markers to label the neurons (SMI312), astrocytes (GFAP) and microglia (Iba-1) on frozen cortical sections collected from adult rat 4 days after receiving needlestick injury with either the A_488_ or MT_488_. The MT_488_, but not A_488_, was visible in neurons (**a**); higher magnification images shown in **b** and **c**, similarly, the astrocytes also appeared to take up the MT_488_ but not A_488_ (**d**); higher magnification images shown in **e** and **f**. In contrast, both A_488_ and MT_488_ were taken up by microglia (**g**); higher magnification images shown in **h** and **i**. The microglia that taken up A_488_ appear in a more amoeboid-like morphology when compared to the microglia receiving the MT_488_, which had a more ramified phenotype. (Scale bar in **a**, **d** and **g** = 50 μm; **b**, **c**, **e**, **g**, **h** and **i** = 12 μm)

We characterised the cells that are associated with the administered MT_488_ after injury by performing immunofluorescent labelling using markers for neurons (SMI312, phosphorylated neurofilament), astrocytes (GFAP) and microglia (Iba-1) (Fig. 1). We show that MT_488_ was associated with neurons (particularly prevalent in axonal structures labelled with SMI312; Fig. 1a), microglia and astrocytes (labelled with Iba-1 and GFAP; Fig. 1d, g). In control tissue injected with A_488_, none of the neurons and astrocytes was associated with the injected A_488_; however, the A_488_ was shown to be associated with the Iba-1 positive microglia (Fig. 1g). Notably, microglia that had associated with MT_488_ displayed a ramified morphology, while those associating with A_488_ displayed a rounded (or amoeboid) morphology. We also observed that the majority of the MT_488_ was located within cellular structures surrounding the lesion site. In contrast, A_488_ was mainly located within the lesion site along with the cells debris.

### Role of metallothionein-II in modifying microglial response after injury

**Fig. 2:**
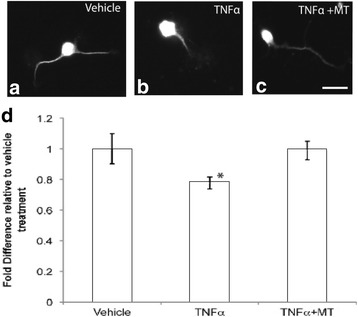
Microglial inflammatory response was inhibitory to axonal outgrowth and this could be modulated (or improved) by extracellular metallothionein-II (MT). **a** shows the immunohistochemistry of neurons in the co-culture using the SMI312 antibody. The neurons treated with TNFα has a shorter process (or axon) when compare to vehicle. The addition of MT to the TNFα treatment has restored this inhibitory effect to a similar level as that receiving vehicle treatment (**a**–**c**). **d** shows the quantitative measurement of the neurite length relative to the vehicle treatment. (Scale Bar in **a** = 12 μm) (*p < 0.05 compared to vehicle treatment and TNFα + MT using ANOVA; *n* = 4 experimental cultures per treatment group; error bars = standard errors of the mean fold difference relative to vehicle treatment)

Given that the uptake of MT_488_ was observed within microglia and that this was associated with an altered cellular morphology, we next examined the effect of exogenous MTII upon reactive microglia, using an in vitro microglia and neuron co-culture model*.* Firstly, we established the effect of activated microglia (TNFα stimulated) upon neurite outgrowth of co-cultured cortical neurons. Measurement of axonal extension 24 h after the plating of cortical neurons demonstrated that axons grown on activated microglia (TNFα-treated) were significantly (*p* < 0.05) shorter than axons grown on microglia treated with saline (vehicle) (Fig. 2a, b). When the axons were plated on microglia that were pre-treated with TNFα and MTII (Fig. 2c), the length of the axons was significantly longer than neurons treated with TNFα only and not significantly different to the saline-treated microglia (Fig. 2d). This demonstrated that the addition of MTII on TNFα-treated microglia ameliorated the inhibitory activity of TNFα-treated microglia, restoring neurite outgrowth to a level similar to that in cells receiving the vehicle treatment (Fig. 2).

### Metallothionein-II acts through LRP1 receptors in microglia

**Fig. 3:**
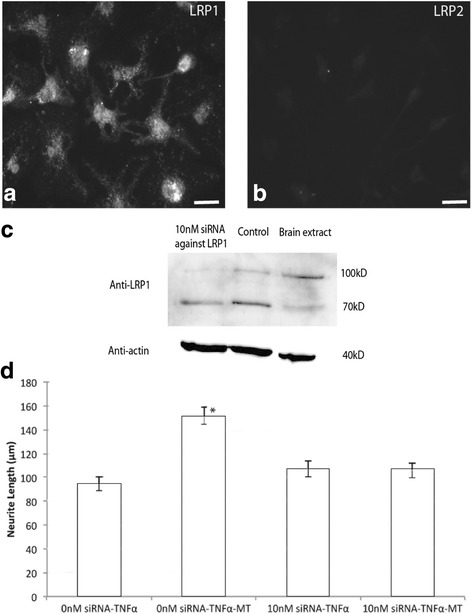
Metallothionein II (MT) alter the TNFα-treated microglia response via LRP1 receptors in microglia and neuron co-culture model. Immunohistochemistry labelling using antibodies against LRP1 and LRP2 demonstrated that cultured microglia express LRP1 but not LRP2 receptors (**a** and **b**). **c** shows the Western blot performed on the protein extracted from microglia culture receiving 10 nM siRNA or no siRNA. In comparison to the no siRNA control, there is a decrease in the expression of LRP1 (both 100kD and 70kD band). The brain extract from rat cortex was also used as an internal positive control for this western blot. **d** shows the quantitative results from the microglia-neuron co-culture with the treatment of siRNA against LRP1 added to the microglia in conjunction with the treatments (TNFα or TNFα + MT). Similar to the result shown previously, in cultures not receiving the siRNA, the neurite length was higher in the microglia pre-treated with MT and TNFα when compare to the treatment with TNFα. The addition of siRNA against LRP1 in the TNFα-treated microglia did not led to significant changes in the neurite length when compared to the TNFα treatment without the siRNA. In contrast, the addition of siRNA against LRP1 did not significantly increase the neurite length. (Scale bar at Figure A = 25 μm) (*p < 0.05 compared to other treatment; *n* = 4 experimental cultures per treatment group, ANOVA; error bars = standard errors of the mean neurite length)

We have previously demonstrated that MTII acts through the LRP2 (or megalin) receptor on neurons to improve regenerative sprouting of injured neurons [9, 10]. Here, we determined if LRP receptors are also involved in the action of MTII upon cultured microglia. We firstly characterised the LRP expression in cultured microglia, and we observed a robust immunolabelling of LRP1 primarily present in the cell bodies (Fig. 3a). In contrast, no LRP2 labelling was observed in the cultured microglia (Fig. 3b).

To determine whether LRP1 is involved in the action of MTII upon cultured microglia, we employed siRNA against LRP1 in the microglia-neuron co-culture model. We first validated the selectivity of the siRNA against LRP 1 receptors in our previous publication [17]. The decreased expression of LRP1 receptors in the presence of siRNA was also confirmed using Western blot (Fig. 3c). We then examined the effect of knocking down LRP1 expression upon the ability of MTII to modulate inflammatory-activated microglia to promote neurite outgrowth. We found that the effect of MTII upon TNFα-stimulated microglia was ablated with reduced LRP1 expression, suggesting that MTII exerts its effect through the LRP1 receptor in vitro (Fig. 3d).

## Discussion

In this study, we first demonstrated that the majority of the exogenously administered MT_488_ and A_488_ associated with the Iba1-labelled microglia/macrophages within close proximity to the needlestick injury in the cortex. The association of both the MT_488_ and A_488_ may be in accordance with the well-described function of microglia to phagocytose foreign material [18]. Using this fluorescence-labelled protein model, we can only demonstrate the association of MTII to microglia in vivo. A further study that involves injecting unlabelled MTII into a transgenic MT-null mice model will be needed to confirm the fate of the injected MTII via post-mortem immunolabelling. However, from what we observed using the fluorescence-labelled MTII, the microglia that had associated with MT_488_ had a ramified phenotype in comparison to those internalising A_488._ Previous studies have suggested the presence of two subtypes of microglia in the CNS; however, recent studies have suggested that these two subtypes could potentially be extremes of a spectrum between the neuro-toxic (M1) to neuro-protective roles (M2) that microglia have in response to an extracellular signal [19]. The changes in microglia phenotype in the presence of MTII observed in this study indicate that MTII plays a role in altering the function of the microglia to a more neuro-protective form rather than a switch in the subtypes. However, further study is needed to clarify the expression of the neuro-protective phenotype markers in the microglia of the brains treated with MT_488_. It should be noted in this experiment model, the amount of MTII injected (1 μL of 2 mg/mL of MTII) is not a significant amount compared to the endogenous MTII concentration in human. The concentration of the MTII used in this experiment model is mainly to allow us to study the cellular association of extracellular MTII, which is the main aim of this experiment. A limitation of this study is the use of the Iba-1 antibody, which labelled both microglia and infiltrating peripheral macrophages; this makes it difficult to specify whether the cells that have internalised the A_488_ and MT_488_ are microglia or infiltrated macrophages or both. To further investigate the direct influence that MTII has on microglia, we utilised a microglia-neuron co-culture model. In this co-culture model, the microglia is pre-conditioned with either TNFα only or TNFα and MTII in serum-free media, which is then replaced with neurobasal media containing 10% serum prior to the plating of cortical neurons; therefore, the addition of the serum-derived MTII will not affect the pre-conditioning on microglia from the treatments, hence the experiment outcome from the co-culture model. Using this model, we have demonstrated that pre-treating the microglia with TNFα (a pro-inflammatory cytokine) led to a decrease in neurite outgrowth and that the addition of MTII abrogated this effect. Hence, this demonstrated that MTII is capable of exerting a specific effect upon TNFα-treated microglia in culture. It has been recently demonstrated that TNFα treatment in primary microglia culture leads to an inflammatory response via suppressing the expression LRP1 [20]. The activation of LRP1 receptors has been shown in various studies to be involved in decreasing the inflammatory response of microglia. It has also been demonstrated that MTII bind to both LRP1 and LRP2 (also known as megalin) receptors in neurons [9, 10, 21]. In this study, we hypothesised that MTII bind to LRP1 receptor on microglia which leads to a decrease in the inflammatory response in microglia through its action on LRP1 receptors. To investigate this, we have utilised siRNA to knockdown LRP1 receptors in our microglia and neuron co-cultures. Our results demonstrated that the knockdown of LRP1 receptor attenuates the effect of MTII in promoting neurite outgrowth after TNFα treatment. This suggests that MTII modulates microglia function via the LRP1 receptor.

This is the first report showing that MTII acts through the LRP1 receptor to alter the microglia response. It has been previously reported that LRP1 receptor activation by an Apolipoprotein-E (Apo-E) mimetic peptide also decreases the microglia inflammatory response via a mitogen-activated protein kinase (MAPK) dependent pathway [22]. Notably, we have reported previously that MTII stimulates the growth of cultured cortical neurons via LRP2 or the megalin receptor [9, 10]. Collectively, this suggests that LRP signalling could be involved in promoting axon regeneration and neural recovery following traumatic injury via multiple cellular pathways. We have previously reported that MTII modulates the behaviour of reactive astrocytes through a JAK/STAT signalling pathway [11]. A recent study had also demonstrated the involvement of LRP1 in microglia activation via modulating the JNK and NF-kappaB signalling pathways [20]. The JAK/STAT, JNK and NF-kappaB have been demonstrated to be important stress kinase pathways involved in microglia inflammatory response [23]. Thus, these pathways could potentially be the downstream pathway for MTII-LRP1 interaction in microglia; however, this will need to be confirmed in future studies.

In this current study, MTII treatment caused a distinct change in the morphology of microglia. In saline-treated animals, microglia displayed a spherical and amoeboid morphology that is proposed to be associated with a reactive and inflammatory phenotype that inhibits neural regeneration (commonly referred to as the M1 phenotype) [24]. However, MTII treatment caused a clear difference in microglial morphology, resulting in ramified structures that resemble the M2 phenotype that supports neural regeneration [24]. Our in vitro studies support the observation that MTII induces a pro-regenerative microglial phenotype in response to TNFα. Collectively, our data clearly demonstrates that MTII activates an LRP1 receptor-dependent pathway in microglia, providing for the first time a mechanistic explanation for the numerous in vivo reports that MTII modulates the microglial response to CNS injury and stress [12–14, 16].

## Conclusions

In conclusion, this study has presented data supporting the neuroprotective role of MTII via modulating the microglia inflammatory response. This study has also identified LRP1 as a receptor for MTII in microglia, which further demonstrates the importance of LRP1 receptors in modulating the microglial response to promote axonal growth after injury. This study has demonstrated that in addition to the direct role that MTII exerts on neurons to promote their regeneration, MTII also indirectly promotes the regeneration (or recovery) of the damaged neurons by improving the extracellular environment (glial response) to support the axon regeneration after injury.

## Abbreviations

Apo-E: Apolipoprotein-E
CNS: Central nervous system
CSPG: Chondroitin sulphate proteoglycans
DMEM: Dulbecco’s modified eagle medium
GFAP: Glial fibrillary acidic protein
IFN-γ: Interferon-γ
LRP: Low-density lipoprotein receptor
MAG: Myelin-associated glycoprotein
MAPK: Mitogen-activated protein kinase
MS: Mouse
MT: Metallothionein
OMgp: Oligodendrocyte myelin glycoprotein
PBS: Phosphate buffer saline
QUIN: Quinolinic acid
RB: Rabbit
TNFα: Tumor necrosis factor-α
